# Effect of Treatment Sequencing of Cytoreductive Nephrectomy on Overall Survival Outcomes in Patients Receiving Immunotherapy for Metastatic Renal Cell Carcinoma

**DOI:** 10.7759/cureus.109868

**Published:** 2026-05-29

**Authors:** Ava Zamani, Hunter Cohn, Ana M Moser, Zoe Michael, Matthew J Krinock, Gloria Fung, Yusra F Shao, Kevin B Ginsburg

**Affiliations:** 1 Department of Urology, Wayne State University, Detroit, USA; 2 Department of Urology, Oakland University William Beaumont School of Medicine, Auburn Hills, USA; 3 Department of Oncology, Karmanos Cancer Institute, Detroit, USA

**Keywords:** cancer therapy, cytoreductive nephrectomy, immunotherapy, metastatic renal cell carcinoma, national cancer database, oncology, overall survival, patient outcomes, treatment sequencing, urology

## Abstract

Introduction

The evolving treatment paradigm for metastatic renal cell carcinoma (mRCC) has prompted ongoing investigation into the role and relevance of cytoreductive nephrectomy (CN). While prior studies have suggested that the sequencing of CN may impact survival in this population, the shift toward immunotherapy (IO)-based regimens limits the applicability of these findings, leaving the role of CN in the IO era incompletely understood. Therefore, we assessed survival outcomes among patients with mRCC treated with IO with or without CN.

Materials and methods

Using the National Cancer Database, we retrospectively identified patients with mRCC treated with IO, with or without CN. We categorized them into one of three groups based on treatment received: IO therapy alone, IO therapy followed by CN (IO/CN), or CN followed by IO therapy (CN/IO). Differences in OS were compared across groups using a multivariable Cox proportional hazards model. Secondary objectives compared postoperative readmission rates, postoperative hospital length of stay, and positive surgical margins among patients undergoing IO/CN and CN/IO.

Results

Among 1,981 eligible patients, 785 (40%) received IO therapy alone, 44 (2.2%) received IO/CN, and 1,152 (58%) received CN/IO. OS differed significantly across the three groups (Wald statistic, p<0.001). Pairwise comparisons demonstrated improved survival for both IO/CN and CN/IO compared with IO therapy (hazard ratio (HR) 0.23, 95% confidence interval (CI) 0.13-0.40, p<0.001; and HR 0.35, 95% CI 0.27-0.43, p<0.001, respectively). However, there was no significant difference in OS between upfront and deferred CN (CN/IO vs IO/CN: HR 0.66, 95% CI 0.39-1.12, p=0.13). Among patients who underwent CN, treatment sequence was not associated with readmission rates (IO/CN vs CN/IO: odds ratio (OR) 1.56, 95% CI 0.16-14.9, p=0.702), hospital length of stay (IO/CN vs CN/IO: incidence rate ratio 1.22, 95% CI 0.88-1.70, p=0.237), or positive surgical margins (IO/CN vs CN/IO: OR 0.91, 95% CI 0.29-2.86, p=0.873).

Conclusions

Among mRCC patients receiving IO, CN was associated with improved OS compared with IO therapy alone, with no difference by treatment sequence. While these findings suggest that the addition of CN to IO may provide a survival benefit in appropriately selected patients, prospective randomized trials are needed to further elucidate and confirm this effect.

## Introduction

The treatment landscape for metastatic renal cell carcinoma (mRCC) has undergone substantial changes over the past two decades [1]. In the early 2000s, management relied primarily on cytokine-based therapies such as interferon-alpha and interleukin-2, with the role of cytoreductive nephrectomy (CN) being debated in the setting of metastatic disease [1]. However, two pivotal randomized trials in 2001 demonstrated a survival benefit for CN followed by interferon-alpha compared with interferon-alpha alone, establishing the role of surgery in appropriately selected patients [2,3].

Shortly after, in 2005, the introduction of vascular endothelial growth factor receptor (VEGFR) tyrosine kinase inhibitors (TKIs), such as sorafenib and sunitinib, marked a paradigm shift. These targeted agents became the standard first-line therapy for treatment-naive mRCC patients, prompting re-evaluation of CN [4-7]. The CARMENA trial demonstrated that sunitinib monotherapy was non-inferior to CN followed by sunitinib, with longer median overall survival (OS) observed in the sunitinib-only arm [5,6]. The SURTIME trial explored treatment sequencing by comparing upfront CN followed by sunitinib with deferred CN after initial sunitinib. The results showed improved survival with deferred CN, suggesting a potential benefit to administering systemic therapy prior to surgery [7].

The transition to immunotherapy (IO) for the treatment of mRCC began in 2015 with the approval of nivolumab as a second-line therapy after VEGFR-TKI treatment failure [1,8]. Since then, IO-based therapies have become the predominant approach, with multiple therapies approved for first-line treatment of mRCC, including ipilimumab plus nivolumab, pembrolizumab plus axitinib, cabozantinib plus nivolumab, and pembrolizumab plus lenvatinib [9].

While the findings of CARMENA and SURTIME have influenced guidelines to no longer recommend routine upfront CN, their applicability to the current era is limited, as IO-based therapies are now used as frontline treatment for mRCC [10-13]. Consequently, the role of CN in the setting of IO remains uncertain and warrants further investigation.

Therefore, we retrospectively reviewed the National Cancer Database (NCDB) to assess the association between survival outcomes and treatment with IO therapy alone, IO followed by CN (IO/CN), or CN followed by IO (CN/IO). We hypothesized that treatment with CN in addition to IO would be associated with improved OS compared with IO alone among patients with mRCC and that the sequencing of CN relative to IO may influence survival and perioperative outcomes.

This article was previously presented as a moderated poster at the 2025 AUA Annual Meeting on April 27, 2025, and as a podium presentation at the 2025 Northcentral Section AUA Meeting on October 15, 2025.

## Materials and methods

Study design and population

We performed a retrospective review of patients with mRCC in the NCDB from 2006 to 2016. The NCDB is a nationwide cancer outcomes database developed through a joint effort between the Commission on Cancer (CoC) of the American College of Surgeons and the American Cancer Society. As a hospital-based registry, it collects data from over 1,500 CoC-accredited cancer programs across the United States and Puerto Rico [14,15].

Inclusion criteria were a diagnosis of mRCC, defined as clinical M stage or American Joint Committee on Cancer clinical stage IV, and receipt of IO with or without CN. The NCDB categorizes systemic therapy variables to include chemotherapy, hormonal therapy, and immunotherapy according to the SEER*Rx Interactive Antineoplastic Drugs Database, but does not capture the specific regimens patients received [16]. Additionally, the NCDB includes all treatments in a patient’s first-line treatment; the IO therapy captured in this study reflects neoadjuvant and adjuvant therapy rather than second- or later-line therapies [17]. Patients were excluded if they had non-RCC histology or received preoperative or intraoperative radiation therapy to minimize confounding from alternative treatments. Eligible patients were classified into one of three groups based on treatment received: IO therapy alone, IO/CN, or CN/IO. This study was deemed exempt from review by the Wayne State University Institutional Review Board.

Study objectives

The primary objective of this study was to compare OS among the three treatment groups, defined as the time from diagnosis to death or to last clinical contact for patients still alive at last follow-up.

Secondary objectives were to evaluate the influence of treatment sequence on perioperative outcomes among patients undergoing surgery (IO/CN vs CN/IO). Outcomes of interest included 30-day postoperative readmission rates, postoperative hospital length of stay, and the presence of positive surgical margins on pathology. These perioperative endpoints were selected to assess short-term surgical morbidity and resection quality. The primary and secondary objectives were selected a priori.

Statistical analysis

Demographic and oncologic characteristics were summarized as medians with interquartile ranges (IQR) for continuous variables and as counts with proportions for categorical variables. Cells with fewer than 11 observations were suppressed due to NCDB Data Use Agreement regulations, with corresponding other cells to avoid back-calculation of the suppressed values. Comparisons across the three treatment groups were made using the chi-squared test for categorical variables and analysis of variance (ANOVA) for continuous variables.

Unadjusted OS probabilities were estimated using the Kaplan-Meier method and compared with the log-rank test. A multivariable Cox proportional hazards model with clustered robust standard errors to account for intra-site correlation was used to estimate the association of treatment type and OS and generate adjusted survival curves. Covariates included in this model were age (continuous), clinical T stage (T1, T2, T3, and T4), race (White vs non-White), insurance type (private, government, and uninsured/unknown), income (zip code median income quartile: <40,227, 40,227-50,353, 50,354-63,332, and >63,332), percent of patients without a high school degree in the zip code (>17.5%, 10.9-17.5%, 6.3-10.8%, and <6.3%), sex (male and female), comorbidity score (0, 1, ≥2), and urban/rurality index (metro, urban, and rural). Group differences were tested using a Wald test, and the following relevant pairwise comparisons were performed: IO vs IO/CN; IO vs CN/IO; and IO/CN vs CN/IO.

For secondary outcomes comparing perioperative outcomes among patients undergoing IO/CN or CN/IO, multivariable logistic regression models were fit to assess postoperative readmission and positive surgical margins, reported as odds ratios (OR), and negative binomial regression was used to evaluate postoperative hospital length of stay, reported as incidence rate ratios (IRR). Both models were adjusted for the same covariates included in the multivariable Cox model for adjusted OS.

Sensitivity analysis

To account for potential immortal time bias in the IO/CN or CN/IO groups, we calculated the median time to the second treatment: 115 days to CN in the IO/CN group and 88 days to IO in the CN/IO group. We then repeated the primary outcome and multivariable Cox model using a four-month (120-day) landmark from diagnosis, excluding patients who died within the first 4 months. This 120-day landmark was applied to all treatment groups. Statistical analysis was performed using Stata version 15.1 (StataCorp, 2017. Stata Statistical Software: Release 15.1. College Station, TX: StataCorp LLC). All statistical tests were set at a significance level of 0.05.

## Results

Demographics

A total of 1,981 patients met the inclusion criteria, of whom 785 (40%) received IO therapy alone, 44 (2.2%) received IO/CN, and 1,152 (58%) received CN/IO. Median age was highest in the IO therapy group (64, IQR 56-72) and lowest in the CN/IO group (57, IQR 50-64). Across all groups, the majority of patients were White. A detailed summary of patient demographics is provided in Table 1.

**Table 1:** Demographics Values are presented as numbers (% for categorical variables) and medians with IQRs for continuous variables. Comparisons were made using the chi-squared test for categorical variables and ANOVA for continuous variables. Per the NCDB Data Use Agreement, cells with fewer than 11 observations were suppressed for confidentiality, along with their corresponding cells, to prevent back-calculation. IO: immunotherapy, CN: cytoreductive nephrectomy, IQR: interquartile range, ANOVA: analysis of variance, NCDB: National Cancer Database

	IO therapy (n=785) (40%)	IO/CN (n=44) (2.2%)	CN/IO (n=1,152) (58%)	p-value
Clinical T stage				
cT1	176 (31%)	-	156 (16%)	
cT2	160 (28%)		342 (35%)	
cT3	142 (25%)	13 (33%)	418 (43%)	
cT4	85 (15%)	-	59 (6.1%)	
Race				0.073
White	678 (86%)	-	1034 (90%)	
Non-White	107 (14%)	-	118 (10%)	
Age	64 (56-72)	60 (54-67)	57 (50-64)	
Income				0.014
<40,227	145 (19%)	-	154 (14%)	
40,227-50353	164 (21%)	-	213 (19%)	
50354-63,332	190 (24%)	-	269 (24%)	
>63,332	278 (36%)	18 (41%)	494 (44%)	
Insurance				
Private	317 (40%)	23 (52%)	771 (67%)	
Government	434 (55%)	-	336 (29%)	
Uninsured/unknown	34 (4.3%)	-	34 (3.9%)	
Education				
>17.5%	171 (22%)	-	181 (16%)	
10.9-17.5%	206 (26%)	-	247 (22%)	
6.3-10.8%	219 (28%)	17 (38%)	356 (31%)	
<6.3%	183 (23%)	-	350 (31%)	
Distance	11.3 (4.7-27.8)	18.3 (7.5-103)	19.8 (8.1-53.6)	0.009
Urban/rural				0.664
Metro	649 (85%)	38 (86%)	930 (83%)	
Urban	96 (13%)	-	160 (14%)	
Rural	20 (2.6%)	-	30 (2.7%)	
Comorbidity score				0.001
0	577 (74%)	33 (75%)	919 (80%)	
1	137 (17%)	-	181 (16%)	
≥2	71 (9.0%)	-	52 (4.5%)	
Sex				
Male	556 (71%)	-	843 (73%)	
Female	229 (29%)	-	309 (27%)	
Year of diagnosis				0.001
2006	34 (4.3%)	-	51 (4.4%)	
2007	22 (2.8%)	-	42 (3.7%)	
2008	23 (2.9%)	-	54 (4.7%)	
2009	16 (2.8%)	-	60 (5.2%)	
2010	18 (2.3%)	-	58 (5.0%)	
2011	17 (2.2%)	-	91 (7.9%)	
2012	23 (2.9%)	-	80 (6.9%)	
2013	72 (9.2%)	-	144 (13%)	
2014	83 (10.6%)	-	167 (15%)	
2015	165 (21%)	-	195 (17%)	
2016	312 (40%)	13 (30%)	210 (18%)	

Overall survival

**Figure 1:**
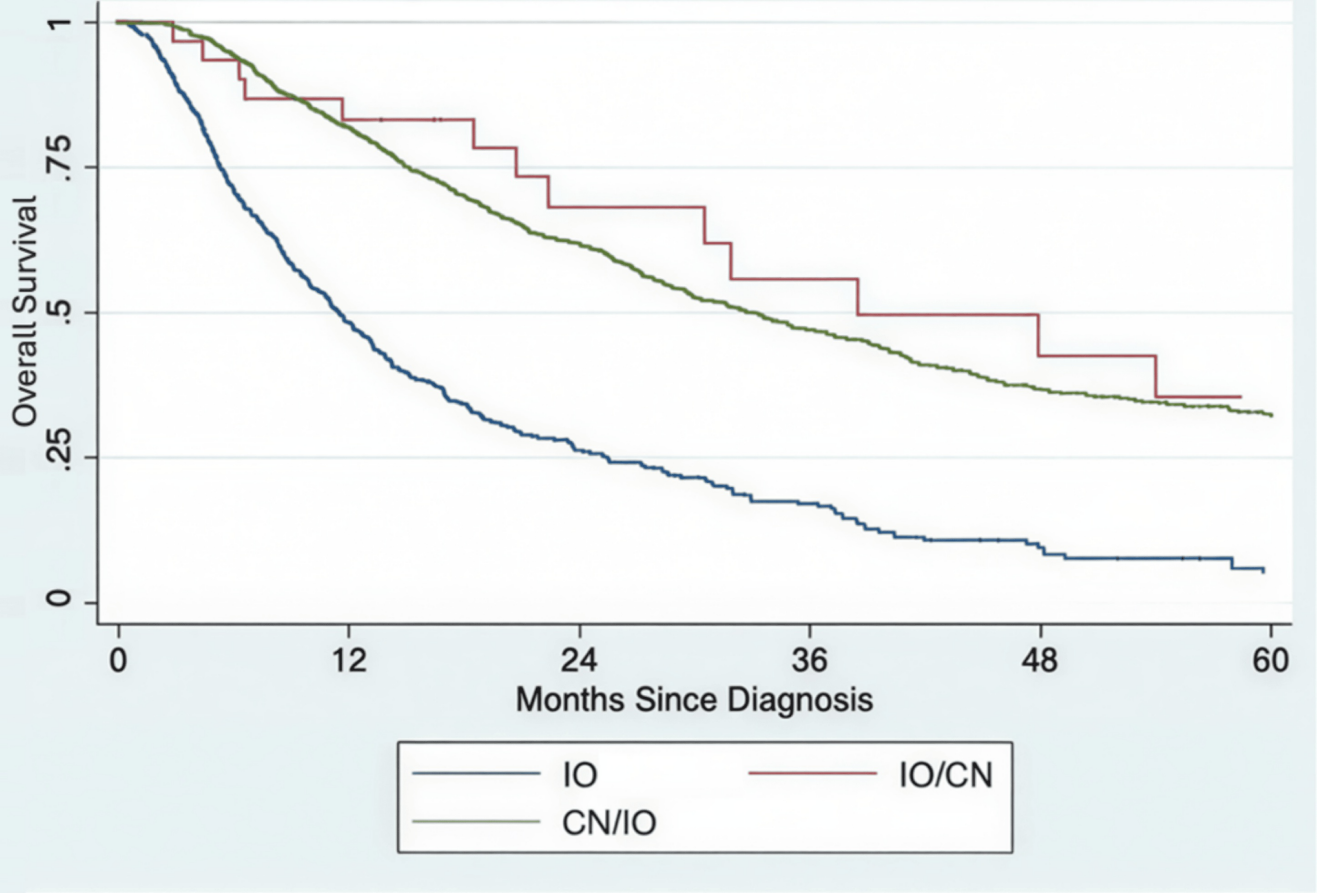
Unadjusted OS estimates Kaplan-Meier curves showing OS among patients with mRCC treated with IO therapy alone, IO/CN, or CN/IO. The IO/CN and CN/IO groups demonstrate improved survival compared with IO therapy. IO-therapy is indicated by the blue line, IO/CN by the red line, and CN/IO by the green line. OS: overall survival, mRCC: metastatic renal cell carcinoma, IO: immunotherapy, CN: cytoreductive nephrectomy

Median unadjusted OS was 11.4 months for IO therapy (95% confidence interval (CI) 10-13 months), 38 months for IO/CN (95% CI 22-not reached), and 33 months for CN/IO (95% CI 30-37 months). OS differed significantly across the three groups (log-rank p<0.001). Pairwise comparisons demonstrated improved survival in both IO/CN and CN/IO compared with IO therapy alone (both p<0.001), without a significant difference between IO/CN and CN/IO (p=0.51). Kaplan-Meier curves demonstrating unadjusted OS are shown in Figure 1.

**Figure 2:**
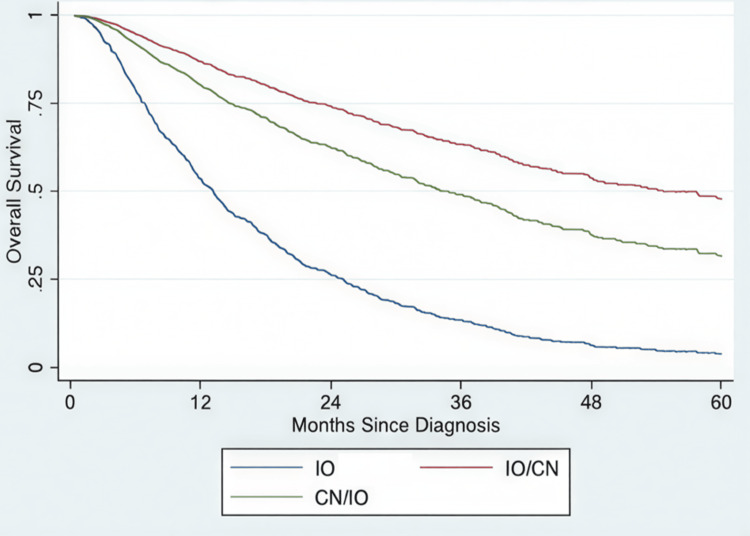
Adjusted OS estimates Multivariable Cox proportional hazards model-derived curves demonstrating OS among patients with mRCC treated with IO therapy alone, IO/CN, or CN/IO. The IO/CN and CN/IO groups demonstrate improved survival compared with IO therapy. IO therapy is indicated by the blue line, IO/CN in red, and CN/IO in green. OS: overall survival, mRCC: metastatic renal cell carcinoma, IO: immunotherapy, CN: cytoreductive nephrectomy

Following adjustment, significant differences in OS persisted across the groups in the multivariable Cox model (Wald statistic, p<0.001). In pairwise comparisons, patients undergoing IO/CN and CN/IO demonstrated significantly improved survival compared with those who received IO therapy alone (hazard ratio (HR) 0.23, 95% CI 0.13-0.40, p<0.001; and HR 0.35, 95% CI 0.27-0.43, p<0.001, respectively). There was no significant difference in survival between CN/IO vs IO/CN (HR 0.66, 95% CI 0.39-1.12, p=0.13). An adjusted OS curve from the multivariable Cox model is shown in Figure 2.

Perioperative outcomes

Among patients who received CN, treatment sequence was not significantly associated with 30-day postoperative readmission rates (IO/CN vs CN/IO: OR 1.56, 95% CI 0.16-14.9, p=0.702), hospital length of stay (IO/CN vs CN/IO: IRR 1.22, 95% CI 0.88-1.70, p=0.237), or positive surgical margins (IO/CN vs CN/IO: OR 0.91, 95% CI 0.29-2.86, p=0.873; Table 2).

**Table 2:** Perioperative outcomes IO: immunotherapy, CN: cytoreductive nephrectomy, OR: odds ratio, IRR: incidence rate ratio, CI: confidence interval

IO/CN vs CN/IO	OR/IRR	95% CI	p-value
Postoperative 30-day readmission	1.56	0.16-14.9	0.702
Postoperative hospital length of stay	1.22	0.88-1.70	0.237
Positive surgical margins	0.91	0.29-2.86	0.873

Sensitivity analysis

The median time from diagnosis to surgery among the IO/CN group was 115 days, and the median time from diagnosis to IO among the CN/IO group was 88 days. Due to concerns about immortal time bias in groups receiving IO/CN and CN/IO, we repeated the primary survival analysis using a four-month landmark corresponding to the median time from diagnosis to surgery in the IO/CN group, which excluded 112 patients. We still observed significant differences in survival among the groups in the multivariable Cox model (Wald statistic, p<0.001), with both IO/CN and CN/IO groups demonstrating improved survival compared with the IO therapy group (HR 0.24, 95% CI 0.14-0.43, p<0.001; and HR 0.37, 95% CI 0.29-0.47, p<0.001, respectively). We did not observe a significant difference between patients undergoing CN/IO and those undergoing IO/CN (HR 0.65, 95% CI 0.37-1.13, p=0.126).

## Discussion

The rapid evolution of therapies for mRCC over the past two decades has created uncertainty and ongoing investigation regarding the role and optimal sequencing of surgery. While prior studies have investigated CN in the context of cytokine and targeted therapies, fewer have evaluated its role in the contemporary IO setting.

Our retrospective analysis partially supported our hypothesis and demonstrated that patients who underwent CN in addition to IO had improved OS compared with those treated with IO therapy alone, suggesting a potential survival benefit of adding CN. This may, in part, reflect improved patient selection, as initial systemic therapy can help identify patients with more favorable or treatment-responsive disease who are more likely to benefit from surgery. Despite this, there was no significant difference in survival between upfront and deferred CN, and perioperative morbidity was comparable between the two CN groups.

These findings should be interpreted in the context of key randomized trials from the VEGFR-TKI targeted-therapy era, such as CARMENA and SURTIME, which helped re-evaluate the role of CN in mRCC. In CARMENA, sunitinib alone was non-inferior to upfront CN followed by sunitinib among patients with mRCC, which challenged the standard of care of upfront CN before systemic therapy [5]. In SURTIME, initial sunitinib therapy followed by deferred CN was associated with improved OS compared with immediate CN followed by sunitinib therapy [7]. While these trials supported a shift away from routine upfront CN in mRCC, their clinical applicability is limited by the current predominance of IO-based therapies. In this context, our findings may suggest that CN is associated with improved survival and may suggest a potential role for surgery in the IO-based therapy era, in contrast with the CARMENA trial findings for the VEGFR-TKI-based regimen era. Additionally, we did not observe a significant OS difference between upfront and deferred CN, in contrast to the findings of the SURTIME trial. This discrepancy may reflect differences in systemic therapy mechanisms, patient selection, and the retrospective nature of the NCDB analysis.

The improved survival observed with the combination of IO and CN may be explained by cellular-level effects in both the neoadjuvant and adjuvant settings [18-22]. As a neoadjuvant therapy, IO may enhance antitumor immunity by utilizing antigens from the primary tumor to activate tumor-specific T cells. The presence of the primary tumor may allow greater expansion and diversification of tumor-specific T cells than administration of IO after tumor removal. These activated T cells help eliminate cancer cells, reducing both microscopic disease and tumor size, thereby potentially preventing disease recurrence [18-21]. From an operative perspective, the reduction in primary tumor size with initial IO can also facilitate resection by increasing the likelihood of complete tumor removal and reducing surgical complexity [18,21].

Surgical tumor resection has been shown to induce a series of inflammatory and metabolic changes that alter cytokine levels [23,24]. While this releases mediators involved in wound healing and pain regulation, it also promotes immunosuppressive pathways that ultimately impair antitumor immunity. Additionally, surgery-related stressors such as hypothermia and blood loss further exacerbate postoperative immunosuppression. In this context, adjuvant IO may help counteract these effects by restoring immune function through inhibition of immunosuppressive pathways, thereby enhancing the activation, proliferation, and activity of antitumor immune cells.

Finally, adjuvant IO following upfront CN may help target minimal residual disease, microscopic tumor cells that persist after surgical resection and are a key contributor to cancer recurrence, thereby reducing this risk [20,22].

These mechanisms of action and the immunologic activity of IO may help explain the improved survival that was observed among patients who underwent CN in our study, as well as similar findings reported in prior studies evaluating CN in combination with IO-based therapies.

Ghatalia et al.’s analysis included a subgroup of 433 patients treated with IO therapy, with or without upfront or deferred CN [25-27]. They reported improved median OS among patients who received CN in addition to IO therapy compared with IO therapy alone (40.2 vs 15.2 months); however, this difference was not statistically significant after multivariable adjustment. There was also no significant difference in survival by the sequence of CN and IO. Similarly, in a cohort of 735 mRCC patients treated with either IO therapy alone, upfront CN, or deferred CN, Gross et al. reported significantly improved median OS among patients who received CN (56.3 months) compared with IO therapy alone (19.1 months), without any difference between the upfront and deferred CN groups [27,28]. Hara et al. compared patients treated with IO therapy with those who received upfront CN followed by IO [29]. In addition to OS, they evaluated less frequently reported outcomes, including progression-free survival (PFS), objective response rate (ORR), and duration of response (DOR). The upfront CN group demonstrated significantly improved PFS (10.8 months vs 3.4 months), OS (38.4 months vs 12.6 months), and ORR (47.6% vs 27.3%), as well as longer DOR (28.3 months vs 13.3 months), compared with those receiving only IO therapy. Notably, the upfront CN group also had three complete responses, whereas the IO therapy group had none. These studies consistently showed an association between adding CN to IO and improved OS, whereas the sequencing of CN does not appear to significantly impact survival.

Much of the evidence to date is retrospective, limiting the ability to draw definitive conclusions. However, multiple prospective trials are underway to provide higher-level evidence and clarity regarding the role and sequencing of CN in the contemporary IO era. The SEVURO-CN trial is among the prospective studies most comparable in design to many existing retrospective analyses evaluating CN with IO therapy for mRCC. As a randomized, multicenter trial, it compares patients treated with IO therapy alone, IO therapy followed by CN, or CN followed by IO therapy. Study endpoints include OS, PFS, ORR, adverse events, and surgical morbidity among patients undergoing CN [30]. The phase III PROBE trial evaluates newly diagnosed mRCC patients treated with systemic therapy with or without deferred CN [31]. All enrolled patients initially receive IO and TKI (IO-TKI) therapy, and following assessment of treatment response, eligible patients are randomized to either continue IO-TKI therapy or undergo CN. Endpoints include OS, treatment response, and post-nephrectomy complications. NORDIC-SUN is also a randomized, multicenter study that compares outcomes in mRCC patients receiving systemic therapy alone vs deferred CN, with systemic options consisting of either IO-TKI or IO therapy [32]. In addition to OS, the trial will assess PFS, ORR, adverse events, and surgical morbidity among patients undergoing CN.

Our study has several limitations. First, its retrospective, non-randomized design introduces the potential for confounding and selection bias. Patients who underwent CN were likely healthier, with more favorable disease characteristics and lower disease burden. This is particularly relevant to patients in the IO/CN group, who likely had IO-stable or responsive disease prior to surgery. It is possible that there were patients in the IO therapy group who initially planned for deferred CN but experienced disease progression or other clinical decline that rendered them ineligible for surgery. Therefore, the IO therapy group may represent patients with a worse prognosis, whereas the IO/CN group may be enriched for IO-responsive patients. Potential unmeasured confounding is also a concern because the NCDB does not capture key clinical variables, including patient performance status, metastatic burden, disease characteristics, IMDC risk classification, and treatment response.

Additionally, the NCDB lacks detail regarding the use of specific IO agents, regimens, and combinations with other therapies, and, as a result, IO treatment was not standardized across patients. These factors remain unadjusted for, potentially affecting the interpretation of our findings. Finally, our IO/CN group size was underpowered at only 44 patients, making it difficult to detect potential differences between sequencing strategies. Given these limitations, our findings should be interpreted with caution and considered hypothesis-generating, rather than practice-changing. Notable strengths of our study include the use of a large, multi-institutional, nationwide cohort, which enabled us to provide real-world data reflecting contemporary clinical practice across diverse patient populations, geographic regions, and practice settings. Furthermore, our analysis provided insight into the clinically relevant question of IO and CN sequencing, as well as perioperative morbidity outcomes, which are underreported in the current literature.

## Conclusions

The role and sequencing of CN in the IO era remain areas requiring further investigation. Our findings align with existing literature demonstrating a survival benefit with the addition of CN to IO therapy, without a significant survival advantage between IO/CN and CN/IO. The comparable perioperative outcomes between both CN groups suggest that treatment sequence does not appear to affect short-term surgery-related morbidity. While these findings support the use of CN with IO, they should be interpreted with caution, as more definitive conclusions await the results of ongoing prospective trials.
